# The effects of scoliosis and subsequent surgery on the shape of the torso

**DOI:** 10.1186/s13013-017-0140-0

**Published:** 2017-11-20

**Authors:** Adrian Gardner, Fiona Berryman, Paul Pynsent

**Affiliations:** 1 The Royal Orthopaedic NHS Foundation Trust, Bristol Road South, Northfield, Birmingham, B31 2AP UK; 2 Department of Anatomy, Institute of Clinical Science, University of Birmingham, Edgbaston, Birmingham, B15 2TT UK

**Keywords:** Scoliosis, Surface topography, Surgery, Shoulders, Axillae, Waist, Normal, ISIS2

## Abstract

**Background:**

Adolescent idiopathic scoliosis (AIS) causes asymmetry of the torso, and this is often the primary concern of patients. Surgery aims to minimise the visual asymmetry. It is not clear how scoliosis makes the torso asymmetric or how scoliosis surgery changes that asymmetry when compared to the distribution of asymmetries seen in a non-scoliotic group of normal controls.

**Methods:**

Surface topography images were captured for a group with AIS both pre-operatively and post-operatively. Identifiable points were compared between the images to identify the effects of AIS on the shape of the torso by looking at the relative heights and distances from the midline of the shoulders, axillae and waist in a two-dimensional coronal view. This was then compared to a previously reported group of normal non-scoliotic children to analyse whether surgery recreated normality.

**Results:**

There were 172 pairs of images with 164 females and 8 males, mean age at pre-operative scan of 13.7 years. The normal group was 642 images (237 females and 405 males) from 116 males and 79 females, mean age of 12.5 years.

The curve patterns seen in the scoliotic group matched the patterns of a main thoracic curve (*n* = 146) and main thoracolumbar curve (*n* = 26). The asymmetries seen in both shoulders, axillae and waist were different between the two different types of curve. Across both groups, the shoulder asymmetry was less than that of the corresponding axillae.

There was a statistically significant reduction in all asymmetries following surgery in the main thoracic group (*p* < 0.001). This was not seen in the main thoracolumbar group, thought to be due to the small sample size. In the main thoracic group, there were statistically significant differences in the asymmetries between the post-operative and normal groups in the shoulders and axillae (*p* < 0.001) but not the waist.

**Conclusions:**

This paper demonstrates quantitatively the range of asymmetries seen in the AIS torso and the degree to which surgery alters them. Surgery does not recreate normality but does cause a statistically significant change in torso shape towards that seen in a non-scoliotic group.

## Background

Within the clinical presentation of adolescent idiopathic scoliosis (AIS), it is common for concern to be raised by both patients and parents around visible asymmetry of the back [1]. This relates to various features including a difference in the height of the shoulders and axillae, inequality of the waist creases and a prominence of one of the scapulae. One of the goals of surgery for AIS is the equalisation of these asymmetries, which translates into improvement in the patient’s self-esteem and life satisfaction [2].

The results of scoliosis surgery are routinely reported as changes in the radiographic Cobb angle [3]. This is a measure of the spinal shape internal to the body rather than the external appearance. There is inherent difficulty in using radiographs as a way of measuring areas and shapes within the body comprised of soft tissue rather than bone. Serial radiography also comes with the price tag of a cumulative radiation dose to the body [4]. Surface topography has been developed as a non-radiation method of documenting the three-dimensional shape of the back. The Integrated Shape Imaging System (ISIS) [5] is now in its second version (ISIS2) [6]. The system analyses a digital photograph of the child’s back which has horizontal lines projected on to it. Fourier transform profilometry is used to create a surface for analysis. The output gives both quantitative and graphical information on the shape for the back in three-dimensions. The use of ISIS2 has been reported previously [6–8].

This paper documents the variability of the relative height of the shoulders, axillae and waist, and also the distance from the midline of the axillae and waist in a group of patients with AIS both pre-operatively and post-operatively. The post-operative values are then compared to previously established normative values for non-scoliotic children [9].

## Methods

Ethical and research governance approval has been obtained for both groups in this study from the NRES committee West Midlands—South Birmingham (11/H1207/10) and the NRES committee East Midlands—Northampton (15/EM/0283).

This analysis is a comparison of two groups. The first is a group of children with AIS who, as part of standard care, have surface topography (ISIS2) measured both before and after surgery as a paired set of images. The second is a group of non-scoliotic children who are part of a longitudinal data collection of surface shape measured using ISIS2 and has been reported on previously [9]. Torso parameters were identified in both groups which were then compared.

All of the scoliotic group had an MRI scan of the whole spine as part of their routine care. Children with neural axis anomalies or other abnormal findings have been excluded from this analysis. None of the study group has been treated in a brace as part of their care. For the majority of subjects, surgery was undertaken using modern posterior based pedicle screw techniques (*n* = 98). An anterior release was used in selected cases for a large stiff curve (*n* = 63). Anterior-only surgery was used selectively for main thoracolumbar curve patterns in the absence of a large compensatory thoracic curve (*n* = 11).

All images in the study were acquired using ISIS2. The degree of spinal curvature in the coronal plane (a two-dimensional measure) was measured with the Lateral Asymmetry parameter from the automated ISIS2 analysis. In this study, a positive number indicated that the scoliosis was convex to the right, and a negative number indicated convex to the left. The ISIS2 images were analysed to find the two dimensional torso points that identify the position of the axillae, shoulders and waist. The axillae points were the most superior points of the posterior axillary folds. The shoulder points were at the superior edge of the torso along a vertical line from the axillae points [10]. The waist points identified were the ‘minimal waist’ [11], which corresponds to the narrowest waist and is the most suitable definition of the waist in a scoliotic population.

**Fig. 1:**
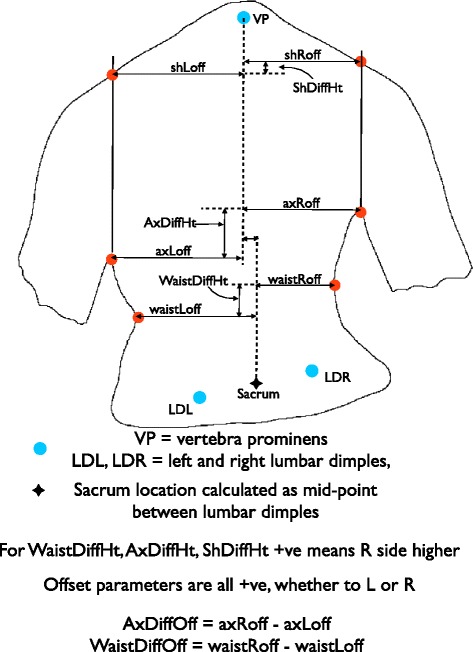
A diagram demonstrating the anatomical points identified and the measurements from the midline for the shoulder, axilla and waist [9]

The positions of the points were then processed to create parameters comparing the two sides of the trunk against each other, Diff Height for a difference in vertical height and Diff Off for a difference in horizontal distance from the midline. This created the parameters Shoulder Diff Height (ShDiffHt), Axillary Diff Height (AxDiffHt) and Waist Diff Height (WaistDiffHt), Axillary Diff Off (AxDiffOff) and Waist Diff Off (WaistDiffOff) (see Table 1 and Fig. 1). Again, a positive number for the measured torso parameter indicated that the right side was higher than the left (DiffHt parameters) or further from the midline than the left (DiffOff parameters).

**Table 1:** A table of the torso parameter and their definitions as shown pictorially in Fig. 1 [9]

Orientation	Torso parameter	Definition
Vertical measurements	ShDiffHt	The difference in vertical height between the shoulder points
	AxDiffHt	The difference in vertical height between the axillary points
	WaistDiffHt	The difference in vertical height between the waist points
Horizontal measurements	axRoff	The horizontal distance from the midline to the right axillary point
	axLoff	The horizontal distance from the midline to the left axillary point
	waistRoff	The horizontal distance from the midline to the right waist point
	waistLoff	The horizontal distance from the midline to the left waist point
	AxDiffOff	The difference between axRoff and axLoff
	WaistDiffOff	The difference between waistRoff and waistLoff

The data on the torso points are presented as data ellipses [12], as this clearly represents the bivariate nature of the data [13]. The layouts are displayed in the same way for each plot for the main thoracic (main thoracolumbar) curves. Pre-operative data are in green (dark green), post-operative data in blue (purple) and the non-scoliotic data in red (orange). The mean point is the solid dot in each colour. The ellipse is the 95% confidence interval about the mean in the respective colour. In the *x*-axis, a positive number is a curve convex to the right. In the *y*-axis a positive number indicates that the right side is higher, or further from the midline, than the left. The box and whisker plots show the data spread of each individual parameter with the median value as the solid bar within the box, which represents the interquartile range. The whiskers from the box represent 1.5 times the interquartile range. Within the box, the dot is the mean value with the 95% confidence interval of the mean as the bars either side.

As there is a difference in the number of pre- and post-operative cases and that of the non-scoliotic group, propensity matching was performed to confirm that this difference did not affect the results.

All analysis was carried out using R [14]. Comparisons of the data were performed with the *t* test for parametric data and the Wilcoxon rank sum test for non-parametric data. Statistical significance was defined as *p* < 0.05.

## Results

The demographic information of both groups is shown in Table 2. In the non-scoliotic group, there have been serial measurements and images captured over 5 years of the same children, with subjects having between 1 and 5 images taken depending on the length of time they have been in the study. Thus, the number of individual images available for analysis is greater than the number of participants. This group consists of 116 males and 79 females. In the scoliotic group, each subject has a pre-operative and post-operative image giving 172 sets of paired data. Neither the time between the pre-operative image and surgery nor between surgery and the post-operative image was normally distributed. Surgery was a median of 346 days after the pre-operative image (IQR 320 days, range 1 to 1211 days). The median time from surgery to the post-operative image was 200 days (IQR 246 days, range 25–1321 days).

**Table 2:** The demographic information of both groups

	Males	Females	Mean age (years)	SD age (years)	Number of images for analysis
Non-scoliotic	405	237	12.5	1.8	642 individual images
Scoliotic	8	164	13.7 (at pre-operative scan)	1.4	172 pairs of pre-operative and post-operative images

The ethnicity in each group was predominantly Caucasian with smaller numbers of participants with either an Afro-Caribbean or Indian heritage. In the scoliotic group, 11% of the total were not Caucasian. In the non-scoliotic group, 3% of the total were not Caucasian.

In the non-scoliotic group, a small curve in the spine in the coronal plane is seen in nearly all of the participants. The major curve was judged to be proximal thoracic (PT) in 21 subjects. There was no curve seen in eight subjects.

As described previously [9], patterns of curve were used to subdivide the data into a main thoracic group with compensatory thoracolumbar curve and a main thoracolumbar curve with compensatory thoracic curve [15]. In the scoliotic group, the largest subgroup had a main thoracic curve with a smaller number with a main thoracolumbar curve. There were no main PT curves. The numbers in each subdivision are shown in Table 3.

**Table 3:** The number in each subdivision of curve type in each group (PT- Proximal thoracic curve, NC- no curve)

	Main thoracic	Main thoracolumbar	Others
Non-scoliotic	387	227	28 (PT and NC)
Scoliotic	146	26	0

**Fig. 2:**
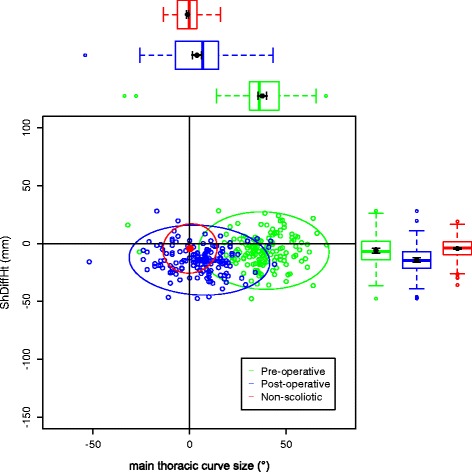
Data ellipses for the main thoracic curve pattern (main thoracic curve) showing ShDiffHt

**Fig. 3:**
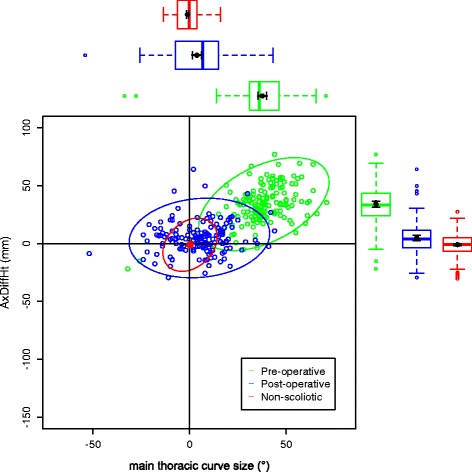
Data ellipses for the main thoracic curve pattern (main thoracic curve) showing AxDiffHt

**Fig. 4:**
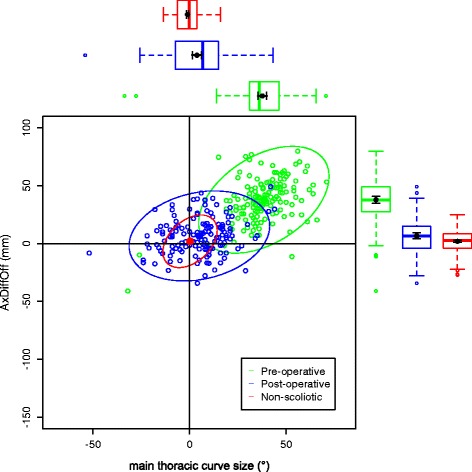
Data ellipses for the main thoracic curve pattern (main thoracic curve) showing AxDiffOff

**Fig. 5:**
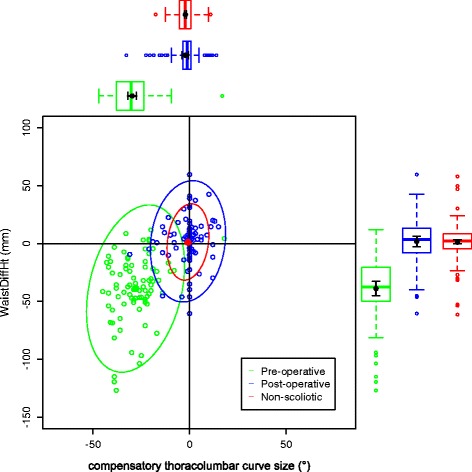
Data ellipses for the main thoracic curve pattern (compensatory thoracolumbar curve) showing WaistDiffHt

**Fig. 6:**
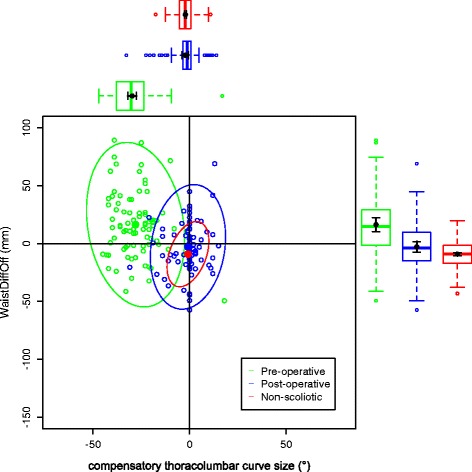
Data ellipses for the main thoracic curve pattern (compensatory thoracolumbar curve) showing WaistDiffOff

**Fig. 7:**
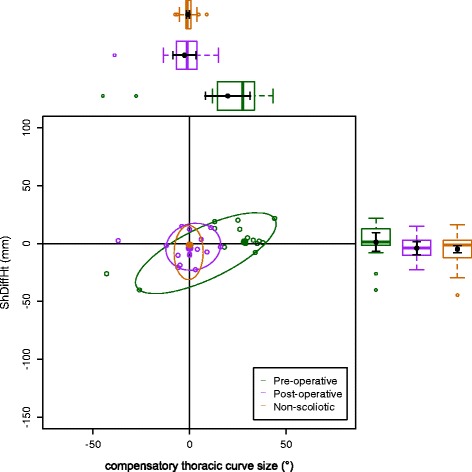
Data ellipses for the main thoracolumbar curve pattern (compensatory thoracic curve) showing ShDiffHt

**Fig. 8:**
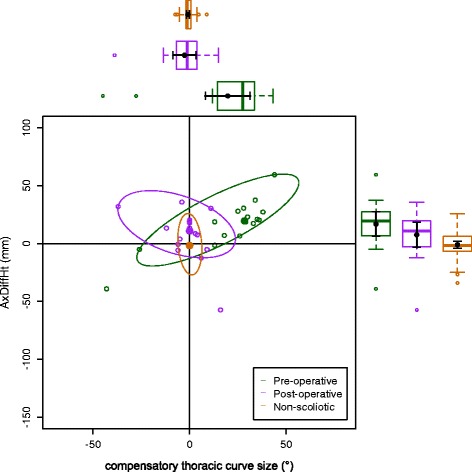
Data ellipses for the main thoracolumbar curve pattern (compensatory thoracic curve) showing AxDiffHt

**Fig. 9:**
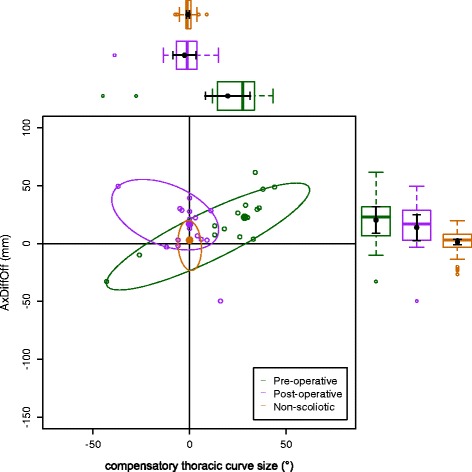
Data ellipses for the main thoracolumbar curve pattern (compensatory thoracic curve) showing AxDiffOff

**Fig. 10:**
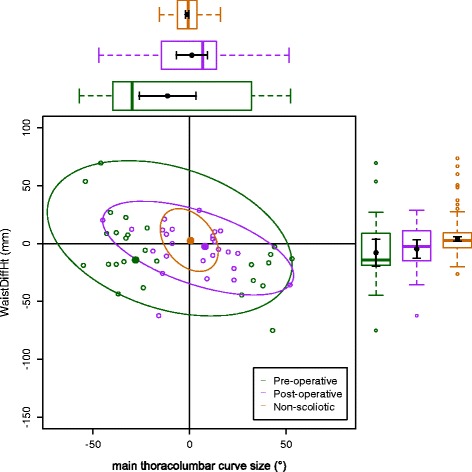
Data ellipses for the main thoracolumbar curve pattern (main thoracolumbar curve) showing WaistDiffHt

**Fig. 11:**
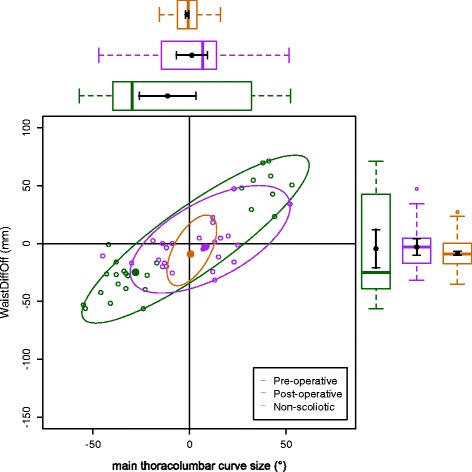
Data ellipses for the main thoracolumbar curve pattern (main thoracolumbar curve) showing WaistDiffOff

The data in the main thoracic curve group were normally distributed. The data in the main thoracolumbar curve group were not normally distributed. Figures 2, 3, 4, 5 and 6 show the data ellipses for the main thoracic curve with compensatory thoracolumbar curve (mean and 95% confidence interval ellipse) and Figs. 7, 8, 9, 10 and 11 show the data for main thoracolumbar curve with compensatory thoracic curve (median and 95% percentile ellipse). The individual data points for the non-scoliotic group are not presented as they obscure the data points of the pre-operative and post-operative groups.

Tables 4 and 5 show the mean (median) values for the parameters in the pre-operative and post-operative groups. The significance in the change from pre-operative to post-operative is also shown. Tables 6 and 7 compare the mean (median) values of the post-operative group to that of the non-scoliotic group.

**Table 4:** A table demonstrating the mean value (and standard deviation) of the parameters measured in the main thoracic pattern for the pre-operative and post-operative scoliotic group with the significance of the change also shown

	Pre-operative	Post-operative	Significance
Curve size (°)	38.5 (13.6)	5.1 (14.6)	< 0.001
ShDiffHt (mm)	−6.1 (13.5)	−14.3 (13.8)	< 0.001
AxDiffHt (mm)	34.1 (16.1)	4.8 (13.8)	< 0.001
WaistDiffHt (mm)	−38.8 (28.6)	1.9 (20.8)	< 0.001
AxDiffOff (mm)	37.9 (18.5)	6.6 (15.6)	< 0.001
WaistDiffOff (mm)	16.3 (28.2)	−2.9 (21.3)	< 0.001

**Table 5:** A table demonstrating the median value (and values of quartile 1 and 3) of the parameters measured in the main thoracolumbar curve pattern for the pre-operative and post-operative scoliotic group with the significance of the change also shown

	Pre-operative	Post-operative	Significance
Curve size (°)	−28.0 (−38.0 to 32.8)	8.0 (−12.8 to 14.5)	0.148
ShDiffHt (mm)	1.5 (−0.8 to 12.5)	−3.9 (−10.9 to 2.8)	0.117
AxDiffHt (mm)	19.6 (6.9 to 27.4)	10.9 (−1.9 to 19.4)	0.044
WaistDiffHt (mm)	−14.1 (−18.7 to 8.4)	−2.5 (−13.9 to 10.7)	0.473
AxDiffOff (mm)	22.8 (7.1 to 31.4)	17.0 (3.1 to 28.5)	0.348
WaistDiffOff (mm)	−24.9 (−37.9 to 39.4)	−3.0 (−16.7 to 4.1)	0.727

**Table 6:** A table showing the statistical analysis of the post-operative group for the main thoracic curve group compared to the non-scoliotic group

	Post-operative scoliosis	Non-scoliotic	Significance of difference
ShDiffHt (mm)	−14.3 (13.8)	−4.3 (8.7)	< 0.001
AxDiffHt (mm)	4.8 (13.8)	−1.0 (9.4)	< 0.001
WaistDiffHt (mm)	1.9 (20.8)	1.4 (13.2)	0.838
AxDiffOff (mm)	6.6 (15.6)	2.0 (9.2)	< 0.001
WaistDiffOff (mm)	−2.9 (21.3)	−9.2 (11.1)	0.013

Non-scoliotic data form Gardner et al. [9]

**Table 7:** A table showing the statistical analysis of the post-operative group for the main thoracolumbar curve group compared to the non-scoliotic group

	post-operative scoliosis	non-scoliotic	significance of difference
ShDiffHt (mm)	−3.9 (−10.9 to 2.8)	−3.9 (8.5)	0.844
AxDiffHt (mm)	10.9 (−1.9 to 19.4)	−2.2 (9.8)	0.004
WaistDiffHt (mm)	−2.5 (13.9 to 10.7)	2.6 (14.0)	0.136
AxDiffOff (mm)	17.0 (3.1 to 28.5)	1.0 (8.4)	< 0.001
WaistDiffOff (mm)	−3.0 (−16.7 to 4.1)	−9.1 (13.1)	0.215

Non-scoliotic data form Gardner et al. [9]

The compensatory curves had no significant difference in effect (see Tables 4 and 5) on the anatomically distant points (for example the effect of the compensatory thoracolumbar curve on the shoulder or axillae points). The waist points and associated trunk imbalance in the main thoracic curve group are due to the effects of the thoracic curve rather than the smaller thoracolumbar curve. This point is further expanded in the ‘Discussion’ section.

Normalising the data for size of torso did not affect the distributions shown in the analysis. The effect of this analysis using a smaller group of non-scoliotic subjects after propensity matching was not appreciably different so the entire cohort of the non-scoliotic group was kept for the analysis.

## Discussion

AIS is a disorder affecting the adolescent spine and is known to come with a ‘psychological burden’. There is a dislike of the asymmetry of the torso and overall body shape that presents with a spectrum of symptoms including mental health disorders [16, 17]. One of the aims of scoliosis surgery is to minimise the visible deformity, improving the symmetry of the torso as safely as possible. In a previous paper, using the same methodology as used here, Gardner et al. [9] have reported the range of normality based on two dimensional torso points in non-scoliotic children. This ‘normal’ group demonstrated that there is a degree of spinal curve in the coronal plane measurable in most children, with differences between the sides of the torso for the shoulder, axillae and waist points. That is, non-scoliotic children are not perfectly symmetrical in the coronal plane and tend to have some spinal curvature, although it is of low magnitude. The data from Gardner et al. [9] acts as a group of normative values to which the AIS group has been referenced.

The AIS group has a larger number of main thoracic curves with compensatory thoracolumbar curves than main thoracolumbar curves with compensatory thoracic curves. This is a similar distribution to that previously reported [15]. The main thoracic curves are mainly convex to the right and an increasing curve is associated with increasing difference between the right and left sides of the torso. The axillae are both more superior (AxDiffHt) and further from the midline (AxDiffOff) on the right in comparison to the left with an increasing scoliosis (Figs. 3 and 4). No effect of an increasing curve on ShDiffHt is seen (Fig. 2). This suggests that the shoulder girdle is compensating for an asymmetry of the underlying torso (demonstrated by the difference in position of the right and left axillae). The independence of movement of the shoulder girdle relative to the torso may well explain why there is moderate to poor correlation of the intraoperative radiographic features of shoulder position to the post-operative shoulder position [18].

The waist is also increasingly asymmetric with an increasing compensatory thoracolumbar curve. As already stated, with an increasing curve, the axillary points become higher and further from the midline on the same side as the convexity of the curve. However, with the waist points, both DiffHt and DiffOff increase in magnitude but in differing directions to each other (Figs. 5 and 6). The reasons for this are unclear but may represent the difference between the relationship of the waist to the spine and the spine to the shoulder girdle. In thoracolumbar curves, the pelvis is the fixed base on which the spine deforms. In thoracic curves the shoulder girdle moves around the already deformed spine.

The effects of the compensatory curve (a thoracic curve on the waist points or a thoracolumbar curve on the axillae and shoulder points) are less clear, although the main thoracolumbar curve has only a small effect on the shoulder and axilla (Figs. 7, 8 and 9). The effect of the main thoracic curve on the waist is more marked and reflects trunk asymmetry caused by a large thoracic curve (Figs. 5 and 6). The effects of the compensatory curve inferior to this thoracic curve are hidden in the effects of the thoracic curve. This is partly due to the mismatch of curve sizes between the main and compensatory curves, with the main curve exerting a relatively larger effect on the shape of the torso. In the main thoracolumbar curve group, a number had a small compensatory thoracic curve. In this circumstance, the overall curve pattern is known to present primarily with waist asymmetry [19]. This could explain the relationship of a thoracolumbar curve on the shoulder and axillae points suggesting that the small thoracic curve exerts a minimal effect.

The number of patients in the main thoracolumbar group is much smaller compared to the number in the main thoracic group. This is the likely reason for the skewed distribution of the data (Figs. 7, 8, 9, 10 and 11) and supports the decision to use non-parametric statistics to analyse this subgroup. With a greater sample size, it would be reasonable to expect a lessening of the effect of the outliers on the average value and a more uniform distribution allowing the use of the mean and 95% predictive confidence ellipse.

Surgical intervention leads to a statistically significant reduction in the size of the scoliosis in both coronal curve patterns (Tables 4 and 5). In the main thoracic group, this is accompanied by a reduction in the amount of asymmetry in the torso at the axillae and waist in both DiffOff and DiffHt (Figs. 3, 4, 5 and 6), and this is statistically significant for all parameters. Interestingly, there is a statistically significant increase in the difference between the left and right sides in ShDiffHt (Fig. 2) with the mean value suggesting that the left is more superior than the right following surgery, a worsening of shoulder height asymmetry, for reasons unknown. The difficulties in achieving balanced shoulders in the post-operative patient remain a challenge [20]. It has been shown that the effect of unbalanced shoulders can reduce over time through other compensatory mechanisms [21]. Reviewing the torso as a whole, surgery is successful in reducing the size of the curve and equalising the shape of the posterior torso.

In the main thoracic group, the ellipses show that surgery improves the torso asymmetry towards that seen in the non-scoliotic group (Figs. 2, 3, 4, 5 and 6). There is still a statistically significant difference in the means for shoulder and axillae points between the post-operative group and the non-scoliotic group (Table 6). However, there is no significant difference in waist position between the post-operative and non-scoliotic groups. The change that occurs following scoliosis surgery is towards the range of asymmetries seen in the non-scoliotic group, although surgery does not completely recreate normality. It is worth noting that in all of the parameters, although the average values are similar, the spread of the data is more dispersed in the post-operative group compared to the non-scoliotic group. Whilst scoliosis surgery changes body shape towards a non-scoliotic population, there is still a difference seen. The answer to the question ‘does scoliosis surgery recreate normality?’ has to be no, but surgery provides a statistically significant change towards a normal shape.

The methodology for the torso points used here is scalar and linear rather than angular as used by Matamalas et al. [22, 23]. The criticism of a non-angular measurement is that it is vulnerable to bias related to differing size between subjects that is not seen in an angular measurement. When all of data presented here was normalised using back length for ShDiffHt, AxDiffHt and WaistDiffHt, axillary width for AxDiffOff or waist width for WaistDiffOff, there were no differences seen in the analysis results and normalisation did not add to the conclusions drawn. Angular measures can be difficult to convert to useful, measurable information in a clinical practice. Linear measures are easy to understand and reproduce and thus are preferred here.

It is noted that the results quoted here represent the position of the torso at the point in time that the post-operative image was taken. With continued growth and then subsequent changes through the ageing process, it is possible that over time, the position described here would change. It would be a valid study to revisit this scoliotic group at 5 years post-surgery to document how the torso has changed over the intervening period.

## Conclusion

This work demonstrates the metrics of trunk asymmetry in a scoliotic group and the effects of scoliosis surgery in reducing these asymmetries. Current surgical techniques do not make the spine straight in the coronal plane, nor do they equalise all asymmetries in the trunk. Surgery can make a statistically significant difference to body shape and when compared to a non-scoliotic group does reduce the size of the torso asymmetries towards the shape of the non-scoliotic torso. Future directions for this work will compare this change in body shape with patient-derived measures of their own deformity, such as the Spinal Appearance Questionnaire [24], to examine what the patients feel about their outcomes from surgery, which previously have been noted to be different from what the surgeon feels has been the outcome [25].
